# Oxidative signaling thresholds shape vulnerability in the central and peripheral nervous systems

**DOI:** 10.1371/journal.pbio.3004032

**Published:** 2026-10-19

**Authors:** Shuji Wakatsuki, Toshiyuki Araki

**Affiliations:** Department of Peripheral Nervous System Research, National Institute of Neuroscience, National Center of Neurology and Psychiatry, Tokyo, Japan; School of Medicine and Health, Technical University, Munich, GERMANY

## Abstract

Neurodegeneration is frequently associated with oxidative stress, yet the mechanisms that convert reactive oxygen species (ROS) into irreversible degenerative signals remain incompletely understood. Neurons in the central and peripheral nervous systems often differ in their responses to injury, but these differences cannot be reduced to a simple contrast between weak and strong antioxidant capacity. This Essay proposes that neuronal vulnerability is shaped by multilayered signaling thresholds comprising ROS production, antioxidant and thiol buffering, redox-sensitive signal detection, molecular gating, and commitment to degeneration, survival, or repair. This framework may help explain selective neuronal vulnerability in neurodegenerative diseases and identify intervention points downstream of global ROS suppression.

## Abstract 2

Neurons in the central nervous system are often more vulnerable to damage during neurodegeneration than neurons in the peripheral nervous system. This Essay argues that differences in how these neurons react to oxidative stress, a major contributor to neurodegenerative disease, could provide a reason why.

## Introduction

Neurodegeneration, a pathological hallmark of many neurological disorders, is often initiated by degeneration of axons and dendrites (collectively termed neurites) [1]. Such neurite degeneration is typically regulated independently from neuronal apoptosis and often precedes neuronal death, leading to detrimental consequences [2]. A striking feature of neurological disorders involving neurodegeneration is that the central nervous system (CNS) and peripheral nervous system (PNS) often exhibit markedly different responses to injury and stress [3–5]. While neurons in the CNS generally show limited regenerative capacity and high susceptibility to degeneration, neurons in the PNS can frequently tolerate substantial stress and retain the ability to regenerate following injury [3–9]. These differences influence disease progression across a wide range of neurological disorders and suggest the existence of distinct molecular mechanisms that determine neuronal vulnerability. However, despite extensive investigation, the basis of this differential susceptibility remains incompletely understood.

Genetic studies have provided important insights into the mechanisms underlying neurodegenerative disorders. For example, more than 30 causative genes have been identified for amyotrophic lateral sclerosis (ALS), a prototypical intractable disease in the field of neurology [10]. However, mutations in any single gene account for only a small proportion of cases of ALS, underscoring the importance of identifying shared pathogenic mechanisms across genetically diverse forms of neurodegeneration. Among the proposed contributory processes, oxidative stress has emerged as one of the most widely implicated in neurodegeneration [11,12]. Increased levels of oxidatively damaged biological components, including proteins, lipids, and nucleic acids, have been reported in numerous neurodegenerative disorders, including Alzheimer’s disease, Parkinson’s disease, Huntington’s disease, ALS, and peripheral neuropathies [11]. However, clinical attempts to broadly suppress oxidative stress using antioxidants have generally shown limited efficacy [13]. These observations suggest that the biological consequences of reactive oxygen species (ROS) are determined not only by their abundance but also by the molecular pathways that interpret and transduce redox signals within neurons.

At the molecular level, these diverse pathological processes are frequently linked by oxidative stress: an imbalance between ROS production and the cellular capacity for detoxification [12]. Excessive or dysregulated ROS signaling can initiate degenerative cascades [11]. A central challenge is therefore to understand how neurons establish molecular thresholds that distinguish adaptive redox signaling from irreversible degeneration. In this Essay, we discuss current perspectives on oxidative stress in neurodegeneration and consider how differential stress tolerance may contribute to the distinct vulnerabilities of the CNS and PNS. We then examine the ZNRF1 pathway as an example of a molecular mechanism that links oxidative stress to neuronal degeneration. Based on this framework, we propose that differences in signaling thresholds, rather than differences in downstream degeneration mechanisms themselves, may be a key determinant of neuronal vulnerability and an underappreciated target for therapeutic intervention.

## Oxidative stress is interpreted through multilayered signaling thresholds

Historically, oxidative stress and ROS have been regarded primarily as mediators of cellular damage associated with aging, cancer, and neurodegenerative disease [14]. However, this view has evolved substantially over the past several decades as physiological functions of ROS have become increasingly recognized [15,16]. In this Essay, we use ‘ROS’ to refer to reactive oxygen species themselves, whereas ‘oxidative stress’ denotes a condition in which ROS production exceeds the cellular capacity for detoxification [12]. This distinction is important because ROS function as signaling molecules under physiological conditions [12,17,18], whereas oxidative stress reflects a disruption of redox homeostasis. ROS can regulate cell proliferation, immunity, autophagy, metabolism, and tissue repair [17–22]; consequently, the presence of ROS does not by itself specify whether a neuron will adapt, degenerate, or die.

The threshold at which induced ROS will produce a subcellular transition can depend on signal amplitude, duration, subcellular location, prior priming, feedback, and the simultaneous engagement of independent pathways. The threshold is therefore an emergent property of a signaling network, not necessarily a fixed ROS concentration. Here, we suggest that four partially overlapping layers are especially relevant for the threshold setting: ROS generation and oxidative input; antioxidant buffering and adaptation; redox-sensitive signal detection and molecular gating; and cellular outcomes, including degeneration, survival/adaptation, and repair/regeneration (Fig 1). Importantly, downstream commitment and non-cell-autonomous response mechanisms need not themselves be directly redox-sensitive. Rather, they can help determine the effective threshold by influencing whether an oxidative perturbation is tolerated, amplified, converted into an irreversible degeneration program, or followed by successful clearance and repair.

**Fig 1:**
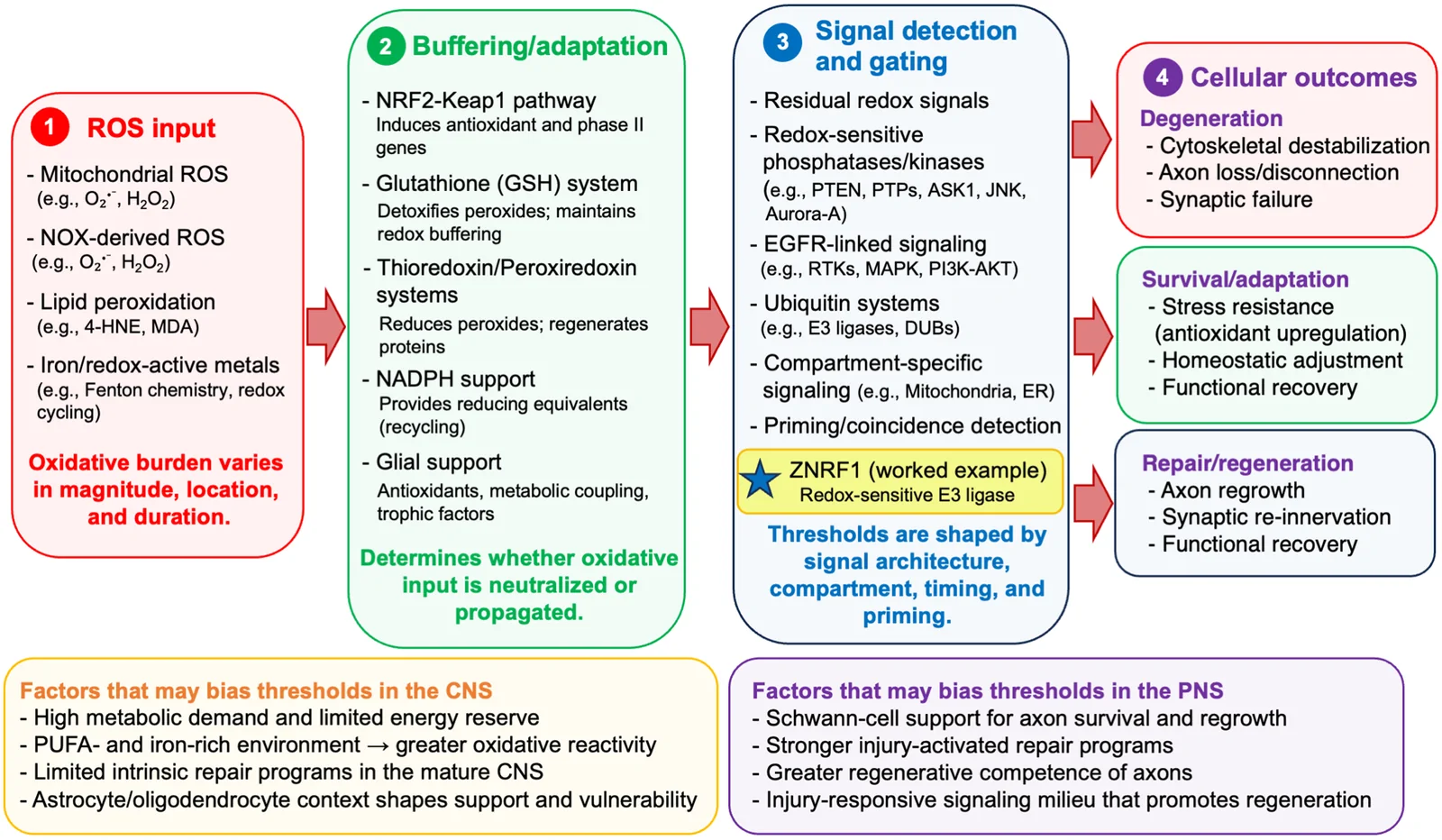
Multi-layer model of oxidative signaling thresholds in neuronal vulnerability. Oxidative inputs arising from mitochondrial and NOX-derived ROS, lipid peroxidation, and redox-active metals vary in magnitude, duration, and subcellular localization. Their biological consequences are shaped first by buffering and adaptive mechanisms, including the NRF2–KEAP1 pathway, glutathione and thioredoxin–peroxiredoxin systems, NADPH-dependent reducing capacity, and glial support. Residual redox signals are further interpreted through redox-sensitive kinases and phosphatases, receptor-linked pathways, ubiquitin systems, compartment-specific signaling, and mechanisms of priming or coincidence detection. Together, these layers determine whether oxidative stress results in degeneration, survival and adaptation, or repair and regeneration. Components of downstream commitment and non-cell-autonomous response mechanisms need not themselves be directly redox-sensitive; rather, they help set the effective threshold by determining whether an upstream redox perturbation is tolerated, amplified, converted into structural degeneration, or followed by successful clearance and repair. These layers are conceptual rather than strictly sequential, and glial and immune cells can act at multiple levels by modifying redox buffering, inflammatory signaling, trophic support, clearance, and regenerative capacity. ZNRF1 provides a mechanistically defined example of a redox-responsive E3 ubiquitin ligase operating within this broader framework. The factors listed for the CNS and PNS represent tendencies that may bias signaling thresholds rather than invariant differences between the two nervous systems. 4-HNE, 4-hydroxy-2-nonenal; AKT, protein kinase B; ASK1, apoptosis signal-regulating kinase 1; CNS, central nervous system; DUB, deubiquitinating enzyme; EGFR, epidermal growth factor receptor; ER, endoplasmic reticulum; GSH, glutathione; H_2_O_2_, hydrogen peroxide; JNK, c-Jun N-terminal kinase; KEAP1, Kelch-like ECH-associated protein 1; MAPK, mitogen-activated protein kinase; MDA, malondialdehyde; NADPH, reduced nicotinamide adenine dinucleotide phosphate; NOX, NADPH oxidase; NRF2, nuclear factor erythroid 2-related factor 2; O_2_•−, superoxide anion; PI3K, phosphoinositide 3-kinase; PNS, peripheral nervous system; PTEN, phosphatase and tensin homolog; PTP, protein tyrosine phosphatase; PUFA, polyunsaturated fatty acid; ROS, reactive oxygen species; RTK, receptor tyrosine kinase; ZNRF1, zinc and ring finger 1.

Threshold logic is widely used in other biological systems to convert continuous and noisy inputs into distinct outcomes. In the DNA-damage response, the duration and temporal pattern of p53 activity can direct recovery, arrest, senescence, or apoptosis even when cumulative signal exposure is similar [23]. Individual p53 target promoters decode amplitude, duration, and frequency using different threshold and filtering properties [24]. And in T cells, distinct pathways downstream of the same antigen receptor show different activation thresholds and dynamic behaviors, allowing signal strength to be translated into graded and digital outputs [25]. These parallels do not imply that neuronal oxidative signaling uses the same molecular circuitry. They illustrate a general design principle: biological specificity can reside in the architecture and dynamics of signal processing rather than solely in the identity or amount of the initiating stimulus. Neurons are especially suitable for such regulation because signals must be integrated across long distances, highly specialized compartments, and long lifetimes. Local redox events in an axon, synapse, mitochondrion, or glial contact may therefore be interpreted differently even within the same cell.

### ROS generation and oxidative input, and antioxidant buffering and adaptation

Increased ROS generation in neurons may be driven by the substantial mitochondrial activity required to meet their high energy demands [4,9]. NADPH oxidase activity also contributes to intracellular ROS levels. ROS buffering, on the other hand, is mediated by two major redox systems: pyridine nucleotides (NAD/NADH; NADP/NADPH) and thiol-based systems, including low-molecular-weight thiol/disulfide couples such as glutathione (GSH/GSSG) and cysteine/cystine [26,27]. In the CNS, mature forebrain neurons can exhibit epigenetic repression of the transcription factor NRF2, whereas astrocytes retain strong NRF2-dependent transcriptional responses [28]. In addition, neuronal activity can induce NRF2-dependent glutathione metabolism genes in astrocytes, illustrating that antioxidant protection in the brain is dynamically distributed across cell types rather than determined solely by the intrinsic antioxidant capacity of neurons [29]. Protein modification by thiol-based redox systems helps determine whether regulatory cysteine modifications remain reversible or progress to persistent oxidation and molecular damage. In human induced pluripotent stem cell-derived neurons, genome-wide CRISPR interference and activation screens identified glutathione peroxidase 4, selenocysteine metabolism, lysosomal function, iron handling, and autophagy as major determinants of survival under chronic oxidative stress; prosaposin, a lysosomal precursor protein required for sphingolipid degradation, was particularly important, as its deficiency produced a neuron-selective pathway that led from lysosomal failure and iron trapping to lipid peroxidation and ferroptosis [30]. Thus, antioxidant capacity is not a single variable but rather a coupled network linking thiol metabolism, organelle quality control, iron homeostasis, and lipid composition.

Neurons in the PNS are generally more resistant to oxidative stress than those in the CNS, but redox regulation in the PNS is not simply biased toward antioxidant protection. Schwann cells can support axons and adopt repair-promoting states after injury, but they can also generate spatially restricted oxidative signals that amplify neuroinflammation; for example, a Schwann cell TRPA1–NOX1 circuit sustains macrophage-dependent oxidative signaling after peripheral nerve injury [31]. NRF2 activation can be protective in experimental peripheral neuropathy [8], yet these observations do not establish that basal NRF2 activity is globally stronger in every PNS neuron or glial state. Collectively, these findings suggest that CNS and PNS tissues may utilize different combinations of neuronal and glial redox-buffering mechanisms, and that these combinations vary according to the type and temporal stage of injury.

### Redox-sensitive signal detection and molecular gating

ROS can influence thiol-based redox systems and induce protein modifications that alter enzymatic activity, protein–protein interactions, localization, and stability. For instance, oxidation of catalytic cysteine residues in protein phosphatases can reversibly suppress their activity, thereby prolonging receptor- and MAPK-dependent signaling [17]. In neurons, reversible oxidation of ERK-directed phosphatases can convert oxidative stress into sustained ERK activity and toxicity [32]. Redox modifications can also activate, rather than inhibit, signaling enzymes. In postmitotic neurons, glutathione S-transferase pi 1-mediated *S*-glutathionylation of Aurora-A at Cys290 promotes its dimerization and autophosphorylation at Thr288, thereby coupling localized redox fluctuations to MEK–ERK signaling and postsynaptic maturation [33]. This example illustrates that redox-sensitive gating can support adaptive or developmental responses as well as degeneration-associated signaling. Thus, the same ROS input can produce different outcomes depending on which phosphatases, kinases, reducing systems, and subcellular microdomains are engaged.

The ubiquitin system is also regulated by redox-mediated modification. For instance, nitrosative or oxidative modification of the cysteine-rich E3 ligase parkin can initially alter and subsequently impair its ubiquitin-ligase activity, linking redox stress to defective protein quality control in models of Parkinson’s disease [34,35]. Another example is the KEAP1–CUL3–NRF2 system, in which oxidative stress-induced modification of KEAP1 inhibits NRF2 degradation. The activity of another E3 ligase, ZNRF1, is regulated by ROS-dependent kinase signaling, leading to degradation of the survival kinase AKT [36,37]. These examples suggest that redox-sensitive ubiquitination can operate as a signal-conversion mechanism rather than merely as a downstream response to cellular damage.

In these examples, ROS-mediated protein modification functions as a molecular gate that requires coincident or sequential subcellular signaling events to translate an increase in ROS into a biological response. Such gates can filter out weak, transient, or spatially inappropriate signals. They can be implemented through priming phosphorylation, scaffold assembly, compartmental recruitment, relief of autoinhibition, or loss of an inhibitory metabolite. The number, order, and subcellular localization of the required events may therefore be as important as the absolute abundance of ROS. This principle underlies our interpretation of the distinct mechanisms of ZNRF1 activation discussed below, but it may also apply broadly to other neuronal stress-response pathways.

### Cellular outcomes, including degeneration, survival/adaptation, and repair/regeneration

The dual leucine zipper kinase (DLK) pathway can serve as a signaling mediator for both protective and degenerative responses in neurons. DLK-dependent signaling can support regenerative transcription after axonal injury, but it can also activate JNK–JUN-dependent degeneration and neuronal death [5,6]. Stress-induced recruitment of DLK to palmitoylated, sphingomyelin-rich vesicular assemblies is required for local kinase activation, illustrating how subcellular organization can create a regulated signaling checkpoint [38]. Moreover, enhanced DLK signaling reduces axonal levels of the survival factors NMNAT2 and Stathmin 2, and synergizes with mitochondrial dysfunction to promote SARM1-dependent degeneration [39]. We consider DLK and the NMNAT2–SARM1 system here as downstream commitment mechanisms rather than primary ROS sensors. Their relevance to the present framework is that they help determine whether an upstream cellular perturbation is converted into irreversible axonal degeneration.

The NMNAT2–SARM1 system provides a particularly clear example of a commitment threshold. Mitochondrial impairment can deplete axonal NMNAT2 and increase the NMN/NAD^+^ ratio [40]. SARM1 senses this metabolic imbalance through its autoinhibitory armadillo repeat domain; once activated, its NAD^+^ hydrolase activity triggers a feed-forward metabolic catastrophe that commits the axon to degeneration [41]. Neuroinflammatory and necroptotic signals can converge on the same execution system by reducing NMNAT2 and Stathmin 2 [42]. In this case, the threshold is determined by the balance among a labile axonal survival factor, metabolite ratios, and an autoinhibited execution enzyme rather than by ROS concentration alone.

These pathways also demonstrate that degeneration and regeneration are not simply opposite ends of a single linear scale. Wallerian degeneration, the prototypical form of neurite degeneration following injury, can facilitate clearance of a disconnected distal axon [43], whereas regenerative competence depends on transcriptional programs, cytoskeletal remodeling, trophic support, and the extracellular environment. A neuron may therefore have a high threshold for somatic death but a low threshold for local axon degeneration, or it may undergo rapid Wallerian degeneration while retaining robust axonal regenerative capacity. This distinction further highlights the importance of non-cell-autonomous support, clearance, and repair in determining the ultimate response to neuronal injury.

## CNS–PNS differences are contextual rather than binary

The CNS and PNS differ in glial composition, vascular and immune interfaces, extracellular matrix, myelination, and the organization of injury responses [3–9]. These differences clearly influence outcome, but they should not be summarized as a universal low threshold in the CNS and high threshold in the PNS. Even within a single sensory neuron, a peripheral axon lesion and a central axon lesion produce different regenerative outcomes. Single-cell profiling of dorsal root ganglia after peripheral and central axon injury has shown that neuronal and non-neuronal populations engage distinct transcriptional and intercellular programs, emphasizing the importance of the local microenvironment [44]. Spatial transcriptomic studies of human neurodegenerative tissue likewise reveal region- and cell-state-specific molecular programs associated with selective vulnerability [45].

Accordingly, the relevant unit of comparison is not ‘the CNS neuron’ versus ‘the PNS neuron’, but a defined neuronal subtype in a defined compartment and injury context, together with its associated glia and immune cells. Apparent CNS–PNS differences may reflect any combination of intrinsic redox buffering, receptor expression, kinase or phosphatase activity, axonal transport, local protein turnover, lipid and iron metabolism, inflammatory signaling, and regenerative support. These variables can shift independently. The threshold framework is valuable only if it is used to organize and test these alternatives rather than to replace them with another binary explanation. Representative pathways illustrate that signaling thresholds can operate at different levels, ranging from antioxidant buffering and injury-signal integration to commitment to axon degeneration (Table 1).

**Table 1:** Representative systems that can establish or shift oxidative signaling thresholds in neurons.

Pathway	Threshold-setting mechanism	CNS evidence	PNS evidence	Direct comparison	Interpretation	Key references
NRF2–GSH	Stress buffering and detoxification; maintenance of thiol redox homeostasis	Mature CNS neurons often show limited cell-autonomous NRF2 activity; astrocyte-supported antioxidant buffering is important	Peripheral neurons and Schwann cells can mount antioxidant and repair-associated protective responses	Limited	Protective threshold	[8,28,29]
DLK–JNK	Injury-signal integration; context-dependent stress-response signaling	Can contribute to degeneration, stress signaling, or cell-death-associated programs depending on context	Also linked to injury responses and regenerative transcriptional programs after axonal damage	Partial	Context-dependent	[6,39,50]
NMNAT2–SARM1	Axon-death commitment controlled by NMNAT2 abundance and the NMN/NAD^+^ metabolic state	Activated in multiple CNS axon-degeneration settings; NMNAT2 loss sensitizes axons	A core executor of Wallerian degeneration and peripheral axon loss	Strong pathway conservation; threshold-setting details remain incompletely compared	Execution threshold	[40–42]
ZNRF1	Direct vs. primed activation of a redox-responsive ubiquitin signaling pathway	ROS–EGFR–ZNRF1 coupling supports degeneration-associated signaling	Additional priming through p38 MAPK–p47^phox^ signaling is required in injured peripheral axons	Mechanistic but limited	Worked example	[36,37,46]

Representative examples are shown; evidence strength differs across pathways, and not all mechanisms have been directly compared between the central nervous system (CNS) and peripheral nervous system (PNS). DLK, dual leucine zipper kinase; EGFR, epidermal growth factor receptor; GSH, glutathione; JNK, c-Jun N-terminal kinase; MAPK, mitogen-activated protein kinase; NAD^+^, nicotinamide adenine dinucleotide; NMN, nicotinamide mononucleotide; NMNAT2, nicotinamide mononucleotide adenylyltransferase 2; NRF2, nuclear factor erythroid 2-related factor 2; ROS, reactive oxygen species; SARM1, sterile alpha and TIR motif-containing protein 1; ZNRF1, zinc and ring finger 1.

Among the pathways linking oxidative stress to neuroaxonal degeneration, the E3 ubiquitin ligase ZNRF1 provides a useful mechanistically defined example [36, 37, 46]. ZNRF1 is expressed in neurons in both the CNS and PNS and participates in axonal degeneration in response to oxidative stress, ischemic injury, and axon severance. ROS-dependent activation of EGFR promotes phosphorylation of ZNRF1, thereby inducing its ubiquitin ligase activity [36]. Activated ZNRF1 ubiquitinates AKT and promotes its proteasomal degradation, thereby releasing GSK3β from AKT-mediated inhibition and activating GSK3β-dependent pathways that destabilize microtubules and drive neurite fragmentation [37]. This cascade illustrates how a reversible redox perturbation can be converted into degradation of a survival signal and, ultimately, an effectively irreversible structural outcome.

The events upstream of ZNRF1 are not identical in the experimental CNS and PNS paradigms examined. In cultured central neurons exposed to oxidative stress, ROS-dependent EGFR activation seems directly coupled to ZNRF1 phosphorylation and activation of the downstream degeneration-associated pathway [36,37]. In injured peripheral axons, by contrast, ZNRF1 activation additionally requires p38 MAPK-dependent phosphorylation of p47^phox^ at Ser345, which promotes NADPH oxidase (NOX)-dependent ROS signaling [46]. We interpret this additional requirement as a two-step gate: injury-associated p38 signaling supplies a permissive state, whereas NOX-derived ROS supplies the activating redox signal (Fig 2). However, this interpretation must be bounded by the evidence. The available studies used different neuronal preparations and stress paradigms and did not generate matched ROS dose–response curves in CNS and PNS neurons. They therefore support a model of distinct activation requirements, but they do not yet establish a quantitatively higher ZNRF1 activation threshold throughout the PNS. Nor do they show that ZNRF1 abundance or intrinsic catalytic capacity is greater in one compartment. Potential factors affecting the differential ZNRF1 activation mechanism include differences in p47^phox^ phosphorylation, NOX assembly, EGFR localization, ZNRF1-associated proteins, phosphatase activity, antioxidant buffering, axonal transport, and the surrounding glial environment.

**Fig 2:**
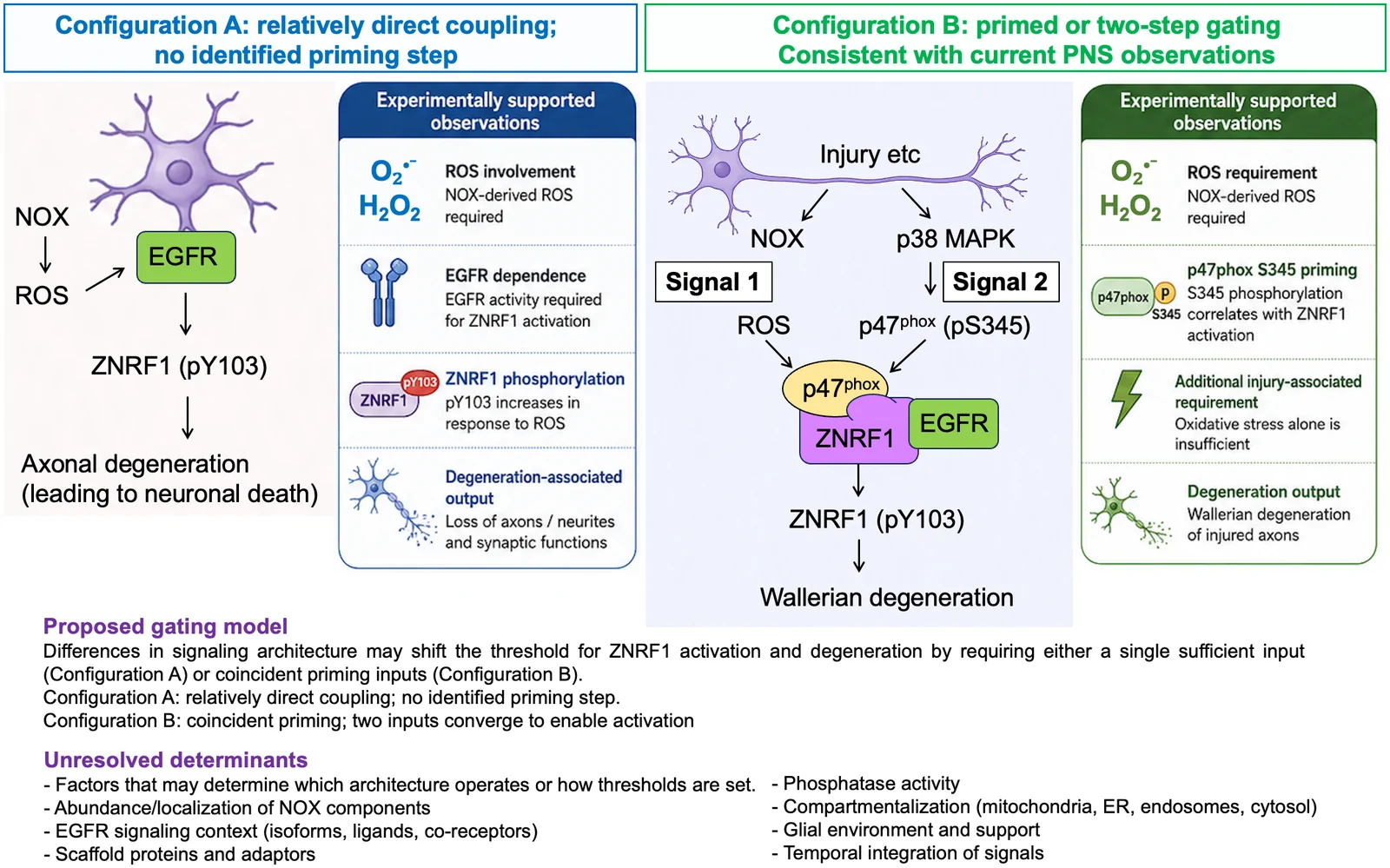
Distinct architectures for ZNRF1 activation: direct coupling and primed gating. Two regulatory configurations consistent with current observations in the CNS and PNS are compared. In configuration A, NOX-derived ROS are relatively directly coupled to EGFR activation, ZNRF1 phosphorylation at Tyr103, and degeneration-associated signaling. In configuration B, observed in injured peripheral axons, ZNRF1 activation requires the convergence of at least two inputs: ROS production and injury-associated priming through p38 MAPK-dependent phosphorylation of p47^phox^ at Ser345. This two-step architecture may reduce inappropriate pathway activation by requiring coincident signals before Wallerian degeneration proceeds. Experimentally supported observations are distinguished from the proposed gating model and from unresolved determinants, including the abundance and localization of NOX components, EGFR signaling context, scaffold and adaptor proteins, phosphatase activity, subcellular compartmentalization, glial support, and temporal integration of signals. These configurations provide a conceptual explanation for how signaling architecture might shift the threshold for ZNRF1 activation, but they do not establish directly quantified or universally applicable CNS–PNS thresholds. These configurations summarize the requirements observed in the experimental paradigms examined to date and should not be interpreted as invariant intrinsic properties of CNS and PNS neurons. CNS, central nervous system; EGFR, epidermal growth factor receptor; MAPK, mitogen-activated protein kinase; NOX, NADPH oxidase; PNS, peripheral nervous system; ROS, reactive oxygen species; ZNRF1, zinc and ring finger 1.

The value of the ZNRF1 example is consequently not that it proves a universal CNS–PNS rule, but that it identifies concrete molecular operations that can create a threshold: direct coupling versus primed activation; regulated kinase access; ubiquitin-dependent removal of a survival signal; and transition to cytoskeletal disassembly. Similar operations may be present in other pathways even when the molecular components differ.

To determine whether the ZNRF1 activation mechanism functions as a signal gate, further experiments will be required. For the ROS-dependent ZNRF1 activation mechanism, local ROS levels should be quantified together with EGFR activation, ZNRF1 Tyr103 phosphorylation, AKT degradation, and neurite fragmentation [37], while the p38–p47phox pathway is independently manipulated [46]. If manipulation of p38 MAPK or p47^phox^ Ser345 alters ROS production but does not change the relationship between local ROS levels and ZNRF1 activation, the p38 MAPK–p47^phox^ pathway would be better regarded primarily as a mechanism controlling ROS production. If the p38 MAPK–p47^phox^ status alters ZNRF1 activation at comparable local ROS levels, this would support a genuine gating mechanism between oxidative input and ZNRF1 activation. Conversely, if ZNRF1 activation remains comparable but AKT degradation or neurite fragmentation differs, the major determinant would lie downstream of ZNRF1 [37]. Thus, the purpose of a matched CNS–PNS comparison is not to assume that the two neuronal populations must differ, but to determine where any experimentally observed difference arises.

## Therapeutic implications: Targeting conversion and commitment

The limited clinical success of broad antioxidant therapies suggests that simply lowering ROS levels may be insufficient and may also interfere with physiological redox signaling [12,13,17,18,20,21]. The threshold framework instead identifies several possible intervention levels. One could increase buffering in a vulnerable cell type, prevent pathological priming or scaffold assembly, inhibit conversion of a redox signal into a degenerative program, or block the execution step after upstream stress has occurred. The optimal level will depend on whether the targeted pathway also supports repair or normal neuronal function.

The DLK and SARM1 fields illustrate both the promise and the risk of this strategy. Pharmacological SARM1 inhibition protects axon structure and function in preclinical models, including chemotherapy-induced peripheral neuropathy [47,48]. By contrast, a phase 1 study of the DLK inhibitor GDC-0134 in ALS identified dose-limiting sensory and ocular adverse events, emphasizing that chronic cell-wide inhibition of a multifunctional stress pathway may have unintended consequences [49]. More selective interference with stimulus-dependent axonal DLK palmitoylation and localization has recently been shown to suppress pro-degenerative signaling while avoiding cytoskeletal effects associated with direct kinase inhibition [50]. These findings support targeting a disease-relevant gating event rather than eliminating an entire signaling system.

ZNRF1 and its upstream activation complex could provide analogous intervention points, but no clinically validated ZNRF1-directed therapy currently exists. Therapeutic development will require demonstration that ZNRF1 is causally engaged in relevant human disease states, identification of druggable interfaces, and definition of the window in which inhibiting degeneration is beneficial rather than delaying clearance of irreversibly disconnected axons. Biomarkers should therefore report pathway engagement—such as ZNRF1 phosphorylation, AKT turnover, SARM1 activity, or compartment-specific redox state—rather than systemic antioxidant capacity alone.

## Conclusions and future directions

Oxidative stress is a common feature of neurodegenerative disorders, but ROS are also essential physiological signals. The key mechanistic problem is therefore how neurons and their surrounding cells distinguish adaptive redox fluctuations from signals that justify irreversible structural destruction. We propose that this decision is governed by multilayered thresholds generated by ROS input, antioxidant and thiol buffering, redox-sensitive molecular switches, coincidence detection, commitment mechanisms, and tissue-level repair capacity. Within this framework, differences between CNS and PNS responses are expected to be contextual rather than absolute. NRF2-dependent neuronal and glial defenses, phosphatase oxidation, ubiquitin signaling, DLK-dependent stress responses, the NMNAT2–SARM1 execution system, and the ZNRF1 pathway can each shift the relationship between oxidative input and outcome. ZNRF1 is a useful worked example because its activation can be formulated as direct coupling or two-step gating, but current evidence does not establish it as the sole or universal basis of CNS–PNS vulnerability.

The next step for the field will be quantitative comparison. Experiments that measure input-output relationships at single-cell and subcellular resolution, under matched CNS and PNS conditions and in the presence of relevant glia, will help to determine whether proposed gates truly alter the threshold for degeneration. More broadly, separating ROS generation from the molecular decisions that interpret ROS may reveal why apparently similar oxidative burdens produce divergent outcomes and may help to identify therapeutic targets that preserve physiological redox signaling while preventing pathological commitment to neurodegeneration.

## Abbreviations

ALS: amyotrophic lateral sclerosis
CNS: central nervous system
DLK: dual leucine zipper kinase
NOX: NADPH oxidase
PNS: peripheral nervous system
ROS: reactive oxygen species
